# *C2* and *CFB* Genes in Age-Related Maculopathy and Joint Action with *CFH* and *LOC387715* Genes

**DOI:** 10.1371/journal.pone.0002199

**Published:** 2008-05-21

**Authors:** Johanna Jakobsdottir, Yvette P. Conley, Daniel E. Weeks, Robert E. Ferrell, Michael B. Gorin

**Affiliations:** 1 Department of Biostatistics, Graduate School of Public Health, University of Pittsburgh, Pittsburgh, Pennsylvania, United States of America; 2 Department of Health Promotion and Development, School of Nursing, University of Pittsburgh, Pittsburgh, Pennsylvania, United States of America; 3 Department of Human Genetics, Graduate School of Public Health, University of Pittsburgh, Pittsburgh, Pennsylvania, United States of America; 4 Department of Ophthalmology and Jules Stein Eye Institute, The David Geffen School of Medicine, University of California Los Angeles, Los Angeles, California, United States of America; 5 University of Pittsburgh Medical Center (UPMC) Eye Center, Department of Ophthalmology, School of Medicine, University of Pittsburgh, Pennsylvania, United States of America; Peninsula Medical School, United Kingdom

## Abstract

**Background:**

Age-related maculopathy (ARM) is a common cause of visual impairment in the elderly populations of industrialized countries and significantly affects the quality of life of those suffering from the disease. Variants within two genes, the complement factor H (*CFH*) and the poorly characterized *LOC387715* (*ARMS2*), are widely recognized as ARM risk factors. *CFH* is important in regulation of the alternative complement pathway suggesting this pathway is involved in ARM pathogenesis. Two other complement pathway genes, the closely linked complement component receptor (*C2*) and complement factor B (*CFB*), were recently shown to harbor variants associated with ARM.

**Methods/Principal Findings:**

We investigated two SNPs in *C2* and two in *CFB* in independent case-control and family cohorts of white subjects and found *rs547154*, an intronic SNP in *C2*, to be significantly associated with ARM in both our case-control (P-value 0.00007) and family data (P-value 0.00001). Logistic regression analysis suggested that accounting for the effect at this locus significantly (P-value 0.002) improves the fit of a genetic risk model of *CFH* and *LOC387715* effects only. Modeling with the generalized multifactor dimensionality reduction method showed that adding *C2* to the two-factor model of *CFH* and *LOC387715* increases the sensitivity (from 63% to 73%). However, the balanced accuracy increases only from 71% to 72%, and the specificity decreases from 80% to 72%.

**Conclusions/Significance:**

*C2/CFB* significantly influences AMD susceptibility and although accounting for effects at this locus does not dramatically increase the overall accuracy of the genetic risk model, the improvement over the *CFH-LOC387715* model is statistically significant.

## Introduction

Age-related maculopathy (ARM), also known as age-related macular degeneration (AMD), is a devastating disorder and a major public health issue. ARM poses one of the greatest threats to vision in the elderly of developed countries and an estimated 1.75 million individuals over 40 years old in the United States suffer vision loss from the disease with an estimated increase to 2.95 million individuals by 2020 [1]. ARM is a degenerative disorder primarily, but not exclusively, affecting the central macular region of the retina. It is characterized by formation of drusen, pigment epithelial changes, atrophic degenerative changes, and formation of choroidal neovascularization.

The etiology of ARM is complex and the disease susceptibility is influenced by both environmental and genetic components [2], [3]. Of modifiable risk factors, the most recognized one is cigarette smoking [4]. In the past couple of years, a light has been shed on our understanding of the genetic susceptibility of the disease [5]. A genome-wide association scan [6] and two targeted searches [7], [8] identified variants in the complement factor H (*CFH,* Entrez GeneID 3075) gene on chromosome 1q32 and two targeted searches [9], [10] identified variants in the poorly characterized *LOC387715* (also known as *ARMS2*, GeneID 387715) gene, as well as in the closely linked *PLEKHA1* (GeneID 59338) and *HTRA1* (GeneID 5654) genes, on chromosome 10q26. Both findings have proven to be robust and the associations of *CFH* and *LOC387715* variants and haplotypes, especially *Y402H* and *S69A*, respectively, have been replicated in multiple cohorts of various nationalities and ethnic backgrounds. This includes mostly samples of white European [11]–[21] and white European American [22]–[37] descent, but also samples of Hispanic origin [38] and samples from Russia [39], India [40], China [41], [42] and Japan [43]–[45]. Negative findings have, however, been reported for the role of *CFH* in Japanese ARM cohorts [46]–[48]. Two more recent studies [49], [50] identified an additional variant (*rs1120638*) in the promoter region of *HTRA1*. This variant is in extremely strong linkage disequilibrium (LD) with the *S69A* variant in *LOC387715*, keeping the debate on the true susceptibility gene in the 10q26 region ongoing [51]–[54]. In the present study, we do not try to distinguish between the genes and variants in this region but use *S69A* as a tagging SNP; given the extensive LD in the region, especially between *S69A* and the *HTRA1* promoter variant, *S69A* can serve as a reasonable proxy for the genetic risk contributed by this region. In fact, a recent fine-mapping effort in this region does suggest that *S69A* is more likely, than the *HTRA1* promoter variant, to be causally responsible for the impact of this locus on ARM [54].

*CFH* is now widely accepted as an important ARM susceptibility gene, harboring variants and haplotypes associated with increased and reduced disease risk. Functional studies suggest that *CFH* inhibits the activation of the alternative complement cascade and complements have been found in the drusen of ARM patients [55]–[59]. It is therefore logical to ask whether other genes involved in the alternative complement pathway may influence the risk. This task was partly tackled by Gold et al. [60] who found ARM-associated variants in the complement component receptor B (*CFB*, GeneID 629) gene and the adjacent complement component 2 (*C2*, GeneID 717) gene on chromosome 6p21. Both genes play a role in complement pathways: *CFB* in the alternative pathway and *C2* in the classical pathway. As was the case for *CFH* and *LOC387715*, this finding also seems robust and has been replicated in two case-control cohorts [27], [61] and one family cohort [61]. However, because of the strong LD across the *C2*/*CFB* region, distinguishing between the genes and identifying true functional variants has proven challenging. Recently two studies [62], [63] reported significant associations between ARM and variants in the complement component 3 (*C3*, GeneID 718) gene on chromosome 19p13. *C3* plays an important role in activation of both the classical and the alternative complement pathways and the plasma complement C3a des Arg levels are significantly elevated in ARM cases compared to controls [64]. A fourth recent study [65] also found ARM associated variants in the *C7* (GeneID 730) and *MBL2* (GeneID 4153) complement pathway genes by complement pathway focused analysis of an earlier genome-wide association scan [6].

In the present study, we investigated four SNPs in the *C2*/*CFB* region, *rs9332739* and *rs547154* in *C2* and *rs4151667* and *rs2072633* in *CFB*, in case-control and family cohorts of white subjects. Only *rs547154*, an intronic SNP in *C2*, was significantly associated with ARM in our data. Subsequently, *rs547154* was used as a tag for this region in multifactor analyses of the joint effect of the three genomic regions (*CFH*, *LOC387715*, and *C2*/*CFB*) on ARM susceptibility.

## Materials and Methods

### Phenotyping, study participants and quality control

Because of the complexity and ambiguity in the ARM phenotype, we have previously defined three affection status models (types A, B, and C) [66], [67]. For clarity we restrict our analyses here to unaffected (or normal) individuals and type A affected individuals. The type A model is our most stringent and conservative diagnostic model and individuals classified as type A ARM affected are clearly affected with ARM based on extensive and/or coalescent drusen, pigmentary changes (including pigment epithelial detachments) and/or the presence of end-stage disease (geographic atrophy [GA] and/or choroidal neovascular [CNV] membranes). Unaffected individuals were those for whom eye-care records and/or fundus photographs indicated either no evidence of any macular changes (including drusen) or a small number (<10) of hard drusen (≤50 µm in diameter) without any other retinal pigment epithelial (RPE) changes. Individuals with evidence of large numbers of extramacular drusen were not classified as unaffected and therefore not included in the analyses. No family member was considered unaffected but was considered of unknown phenotype if not affected with type A ARM.

Using only the subset of white participants, our data include 611 ARM families, 187 unrelated cases and 168 unrelated controls. The ARM families consist of 1,524 genotyped individuals (569 males and 955 females) and, in terms of genotyped affected relative pairs, the families include total of 501 sib pairs, 7 half sib pairs, 60 cousin pairs, 13 parent-child pairs, and 38 avuncular pairs; Pedstats (version 0.6.8) [68] was used to get summary counts of the family data. See Table 1 for other characteristics of the subjects. Before analyzing the family data, PedCheck (version 1.1) [69] was used to check for Mendelian inconsistencies. Since it can be extremely difficult to determine who exactly has the erroneous genotype within small families [70], we set genotypes of problematic markers to missing for every individual within each family containing a Mendelian inconsistency; this needed to be done for only one SNP (*rs859705* in part 3) in one family.

**Table 1:** Samples sizes and other characteristics of the data.

		Family data		Case-control data	
		Type A	not Type A	Cases (Type A)	Controls
Number of genotyped individuals					
	Females	690	265	113	87
	Males	405	164	74	81
	Total	1095	429	187	168
Mean age (SD)					
	Females	77.7 (7.3)	73.4 (12.9)	78.6 (7.0)	71.3 (10.2)
	Males	77.0 (7.1)	73.3 (11.5)	79.8 (6.0)	74.6 (9.4)
	Total	77.4 (7.2)	73.4 (12.4)	79.1 (6.6)	72.9 (9.9)
Cigarette smokers (%)					
	Females	37	35	43	34
	Males	61	50	55	42
	Total	46	41	48	38
GA (%)					
	Females	56	…	55	…
	Males	52	…	58	…
	Total	54	…	56	…
CNV (%)					
	Females	70	…	64	…
	Males	71	…	69	…
	Total	70	…	66	…

GA = geographic atrophy

CNV = choroidal neovascular membranes

SD = standard deviation

Informed consent was obtained from all participants under research protocols that have been reviewed and approved in accordance with the Declaration of Helsinki and the Guidelines for Human Subjects Protection issued by the Office of Human Subjects Research (National Institutes of Health) by the University of Pittsburgh IRB (#9506133) and the University of California–Los Angeles IRB (#10-06-096-01).

### Genotyping

The variants: *rs9332739* (*E318D*) and *rs547154* (*IVS10*) in *C2*, and *rs4151667* (*L9H*) and *rs2072633* (*IVS17*) in *CFB*, were genotyped using 5′ exonuclease Assay-on-Demand TaqMan assays (Applied Biosystems Incorporated). Amplification and genotype assignments were conducted using the ABI7000 and SDS 2.0 software (Applied Biosystems Incorporated, Foster City, CA). The variant *rs1061170* (*Y402H*) in *CFH* and the variant *rs10490924* (*S69A*) in *LOC387715* were genotyped using RFLP techniques. The primers, annealing temperatures and restriction endonuclease for each assay were: 5′-TCTTTTTGTGCAAACCTTTGTTAG-3′ (F), 5′-CCATTGGTAAAACAAGGTGACA-3′ (R), 52°C, NlaIII for *Y402H* in *CFH*; 5′-GCACCTTTGTCACCACATTA-3′ (F), 5′-GCCTGATCATCTGCATTTCT-3′ (R), 54 °C, PvuII for *S69A* in *LOC387715*. For all genotyping conducted for this research, double-masked genotyping assignments were made for each variant and compared; each discrepancy was addressed using raw data or by re-genotyping. Genotype efficiency for the *C2/CFB* SNPs ranged from 93% to 96% and 88%–90% for the two previously published *CFH* and *LOC387715* SNPs.

### Association analyses and LD estimation

#### Case-Control data

Using the set of unrelated cases and controls, SNP-disease allelic and genotypic associations were tested using the Fisher's exact test as implemented in R (version 2.2.1) [71]. For significantly associated SNPs the strength of the association was estimated by crude odds ratios (ORs) and population attributable risks (PARs). To calculate the PARs we used the general formula: PAR = P_f_(OR−1)/(1+P_f_(OR−1)), where P_f_ is the prevalence of the risk or protective factor (genotype) in the general population as estimated from the controls. The ORs were calculated using logistic regression models in R. Confidence intervals (CIs) for the ORs and PARs were derived using the asymptotic normal distribution of ln(OR) and ln(1-PAR), respectively. Haplotypic associations of 2- and 3-SNP moving window haplotypes in the *C2*/*CFB* locus were evaluated using the haplo.cc function of the haplo.stats package (version 1.2.2) [72] of R. This function implements a score test for global test of association between binary traits and haplotypes and accounts for ambiguous linkage phase by the EM algorithm; empirical P-values were generated using 10,000 replicates. Allele and genotype frequencies were estimated by direct counting and deviations from Hardy-Weinberg equilibrium (HWE) were tested, in cases and controls separately, using the exact test as implemented in R Genetics package (version 1.2.1) [73]. Haploview (version 3.32) [74] was used to estimate the LD across the *C2*/*CFB* region, both D' and r^2^ were calculated separately in cases and controls.

#### Family data

When incorporating cases from the families into the analyses, the CCREL method (version 0.3) [75] was used to test SNP-disease allelic, genotypic and 2- and 3-SNP haplotypic associations. The CCREL method permits testing for association with the use of related cases and unrelated controls simultaneously and, briefly, it accounts for biologically related subjects by calculating an effective number of cases such that individuals are assigned weights that are used to construct a composite likelihood, which is then maximized iteratively to form likelihood ratio tests. For the CCREL analyses, type A-affected family members were assigned the phenotype “affected”, unrelated controls the phenotype “normal” and family members not affected with type A ARM the phenotype “unknown”.

### Multifactor and gene-gene interaction analyses

To build predictive models of the genetic risk of ARM contributed by the *CFH*, *LOC387715*, and *C2*/*CFB* loci, we applied both logistic regression and the new generalized multifactor dimensionality reduction (GMDR) method (version 0.7) [76]. The GMDR method, unlike the original MDR method [77], permits adjustment for covariates and better handles data with unequal numbers of cases and controls, and can be used to analyze both qualitative (e.g. binary) and quantitative traits via different link functions. Both methods only handle unrelated individuals. Therefore, to make use of more of our data, we combined one type A affected person picked at random from each of the 611 ARM families with the data of unrelated cases and controls. We consider this to be appropriate to do since the association results suggest the effects of the genes to be similar in both groups.

#### Logistic regression

For each pair of loci, we first followed the modeling strategy proposed by North et al. [78] for two-factor genetic risk models. A series of logistic regression models were fitted to the data in order to find a parsimonious model for the joint effects of each pair of loci. Models allowing for additive effects (ADD1, ADD2, and ADD-BOTH), models incorporating dominance effects (DOM1, DOM2, and DOM-BOTH), and three interaction models (ADD-INT, ADD-DOM, and DOM-INT) were fitted. We fit three-factor models of the joint effect of all three loci and test, using a likelihood ratio test (LRT), whether accounting for the protective effects at *C2*/*CFB* significantly improves the fit of a model with *CFH* and *LOC387715* effects only. Since, for each pair of loci, the two-factor analyses implicated additive models as the most parsimonious and to keep the number of parameters as small as possible we only fit three-factor additive models without interaction (ADD1, ADD2, ADD3, ADD12, ADD13, ADD23, and ADD123). The models are compared by the Akaike information criterion (AIC); the most parsimonious model has the lowest AIC and a model is considered to provide a significantly better fit to the data if it has AIC more than 2 units lower than the comparison model [78]. Details regarding coding of genotypes in the models are available in the supporting information (Text S1).

#### GMDR

Just as in the case of logistic regression, when using the GMDR method, one needs to be aware of the risk of overfitting, especially in the case of small sample sizes. The GMDR method, however, uses cross-validation to guard against overfitting. We applied the method to our data in order to identify three-locus genotypes associated with increased and decreased disease risk. For comparison we also present and discuss the *CFH* and *LOC387715* two-factor model. We performed both crude analysis and analyzed the data while adjusting for age, gender, and cigarette smoking. We used 5-fold leave-one-out cross-validation and exhaustive search of all possible one- to three-locus models in the GMDR analyses. In the adjusted analysis age (in years) was the age at the time blood was drawn (i.e. DNA donated), and cigarette smoking was a binary variable (ever vs. never smoked). The smokers smoked on averaged 40.45 (standard deviation [SD] 32.96; range 0.23–207.00) pack-years (years×packs/day smoked) of cigarettes. The sample in the adjusted analysis includes fewer observations (557 cases and 118 controls fully typed at all three SNPs) than the sample in the unadjusted analysis (640 cases and 142 controls fully typed at all three SNPs) because of missing information. We compared both the sensitivity = TP/(TP+FN) and the specificity = TN/(TN+FP) of the models, where TP = number of true positives, TN = number of true negatives, FP = number of false positives, and FN = number of false negatives. As a single measure of the accuracy of the models we used the balanced accuracy = (sensitivity+specificity)/2 rather than the accuracy = (TP+TN)/(TP+TN+FP+FN) because number of cases and controls is unequal. The average sensitivity, specificity, and balanced accuracy over the testing sets of all five cross-validations are reported. As a measure for the appropriateness of the models, the sensitivity, specificity, balanced accuracy, and P-value are reported for all models when applied to the whole dataset.

### Interaction with cigarette smoking

In a logistic regression framework we tested, using a LRT and the combined data of unrelateds and one type A affected from each family, whether cigarette smoking interacts with the SNPs at the three genes. The genotypes were coded in additive way, as in the logistic regression analysis above, and cigarette smoking as ever vs. never smoked.

## Results

### Results of association analyses

**Figure 1:**
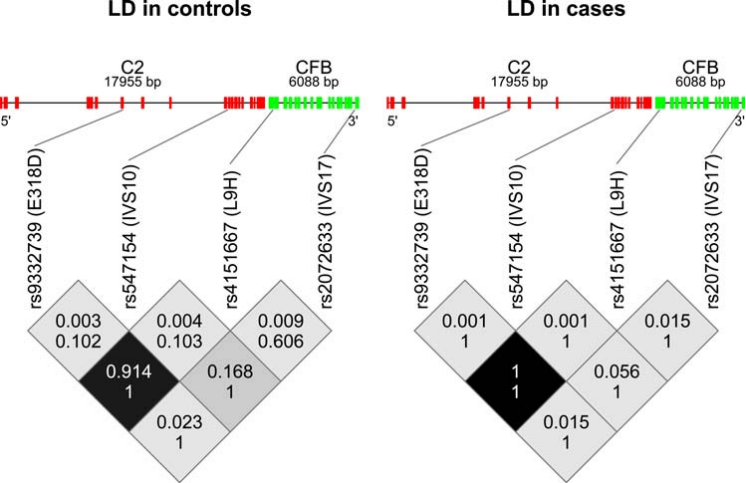
Linkage disequilibrium (LD) across the *C2/CFB* region in unrelated cases and controls. The darker the boxes the higher the r^2^. The top number in each box is r^2^ and the bottom number is D'. Locations of the SNPs within the genes are shown. Red lines/boxes show the locations of exons in *C2* and green lines/boxes the locations of exons in *CFB*.

The genotype distributions of the 4 SNPs typed in *C2* and *CFB* and the *Y402H* variant in *CFH* and the *S69A* variant in *LOC387715* are in HWE in both our cases and controls (Table 2). Of the 4 SNPs typed in the *C2*/*CFB* region, only *rs547154*, an intronic SNP in *C2*, is significantly associated with ARM (Table 2) in both our case-control (P-value of genotypic test 0.00007) and family data (P-value of genotypic test 0.00001), which is also significant after adjusting for multiple testing of 4 tests (Bonferroni corrected 0.05 significance level is 0.0125). The haplotypic association tests show that haplotypes spanning the entire *C2*/*CFB* locus are significantly associated with ARM (Table 2). Although LD between *rs547154* and the SNPs in *CFB* (Figure 1) is not strong, in neither cases nor controls, these results are not sufficient to rule out either *C2* or *CFB* as an ARM candidate gene, because of limited number of SNPs investigated. Individuals carrying the protective allele at *C2* are at 0.22 (95% CI 0.10 to 0.48) times less risk of having ARM compared to controls as estimated with a crude OR. The corresponding PAR is –18% (95% CI –28% to –8%). Detailed results of marginal association of Y402H in CFH and S69A in LOC387715 are in Table 2 and the supporting information (Text S2).

**Table 2:** Association results for *C2/CFB* variants, *Y402H* in *CFH*, and *S69A* in *LOC387715*

					P-value for test									
							Single SNP in				Moving window haplotypic test			
			MAF in		HWE in		CCREL		Exact test in unrelateds		CCREL		Global test in unrelateds	
SNP (Location)	Gene	MA	Cases	Controls	Cases	Controls	Allelic	Genotypic	Allelic	Genotypic	With 2 SNPs	With 3 SNPs	With 2 SNPs	With 3 SNPs
*rs9332739 (E318D)*	*C2*	*C*	0.027	0.033	1.000	0.157	0.26542	0.37187	0.66583	0.90278	0.00088	0.00076	0.00020	0.00000
*rs547154 (IVS10)*	*C2*	*T*	0.025	0.096	1.000	0.365	0.00010	0.00001	0.00011	0.00007	0.00071	0.00131	0.00020	0.00030
*rs4151667 (L9H)*	*CFB*	*A*	0.028	0.036	1.000	0.185	0.19863	0.27610	0.66609	0.81796	0.38067	…	0.27480	…
*rs2072633 (IVS17)*	*CFB*	*A*	0.330	0.393	0.499	0.104	0.64767	0.07003	0.09299	0.05780	…	…	…	…
*rs1061170 (Y402H)*	*CFH*	*C*	0.621	0.348	0.615	0.288	<0.00001	<0.00001	6.3×10^−12^	7.7x^−11^	…	…	…	…
*rs10490924 (S69A)*	*LOC387715*	*T*	0.470	0.200	0.272	0.075	<0.00001	<0.00001	4.2x^−^10^−13^	8.2x^−11^	…	…	…	…

MA = minor allele

MAF = minor allele frequency

HWE = Hardy-Weinberg equilibrium

The haplotypic P-values correspond to the haplotypes of the SNP in the same row as the P-value and the next

one or two SNPs for the ‘With 2 SNPs’ and ‘With 3 SNPs’ P-values, respectively

Genotype counts in unrelated cases and controls are available in Table S1

### Results of multifactor analyses

#### Logistic regression

First we fitted two-factor genetic risk models for each pair of loci and found that an additive model without interaction was the most parsimonious in all cases (Table 3). Three-factor additive model was then fitted in order to test whether the three-factor model provided better fit to the data than any two-factor models (Table 4). The three-factor model of *CFH*, *LOC387715*, and *C2* SNPs coded in additive fashion was the most parsimonious and fitted significantly better (P-value of LRT 0.002) than the next-best model (which modeled *CFH* and *LOC387715* additive effects only).

**Table 3:** Results of fitting two-factor logistic regression models.

Two-factor model			
CFH (Factor 1) and LOC387715 (Factor 2)		AIC	AIC difference
	ADD1	702.6	68.2
	ADD2	699.0	64.5
	**ADD-BOTH**	**634.5**	**0.0**
	DOM1	704.0	69.5
	DOM2	698.6	64.2
	DOM-BOTH	634.9	0.5
	ADD-INT	636.1	1.6
	ADD-DOM	634.5	0.0
	DOM-INT	636.4	1.9
CFH (Factor 1) and C2 (Factor 2)			
	ADD1	716.3	8.7
	ADD2	764.9	57.3
	**ADD-BOTH**	**707.6**	**0.0**
	DOM1	717.6	10.0
	DOM2	764.9	57.3
	DOM-BOTH	709.0	1.5
	ADD-INT	707.7	0.1
	ADD-DOM	709.9	2.4
	DOM-INT	709.9	2.4
LOC387715 (Factor 1) and C2 (Factor 2)			
	ADD1	729.1	13.2
	ADD2	783.7	67.8
	**ADD-BOTH**	**715.9**	**0.0**
	DOM1	729.2	13.3
	DOM2	783.7	67.8
	DOM-BOTH	716.0	0.1
	ADD-INT	717.9	2.0
	ADD-DOM	718.3	2.4
	DOM-INT	718.3	2.4

Detailed model definitions are given in the ‘Materials and Methods–Multifactor and interaction analyses‘ section and Text S1. AIC difference is the difference from the AIC of the best fitting model. Most parsimonious model is in bold. Model with best fit (lowest AIC) has AIC difference = 0.

**Table 4:** Results of fitting three-factor logistic regression models.

Model	AIC	AIC difference
ADD1	685.5	71.2
ADD2	682.8	68.6
ADD3	728.3	114.1
ADD12	622.1	7.9
ADD13	677.8	63.6
ADD23	669.2	55.0
**ADD123**	**614.2**	**0.0**

Factor 1 is *Y402H* in *CFH*, Factor 2 is *S69A* in *LOC387715,* and Factor 3 is *rs547154* in *C2*. Detailed model definitions are given in the ‘Materials and Methods–Multifactor and interaction analyses‘ section and Text S1. AIC difference is the difference from the AIC of the best fitting model. Most parsimonious model is in bold. Model with best fit (lowest AIC) has AIC difference = 0.

#### GMDR

**Figure 2:**
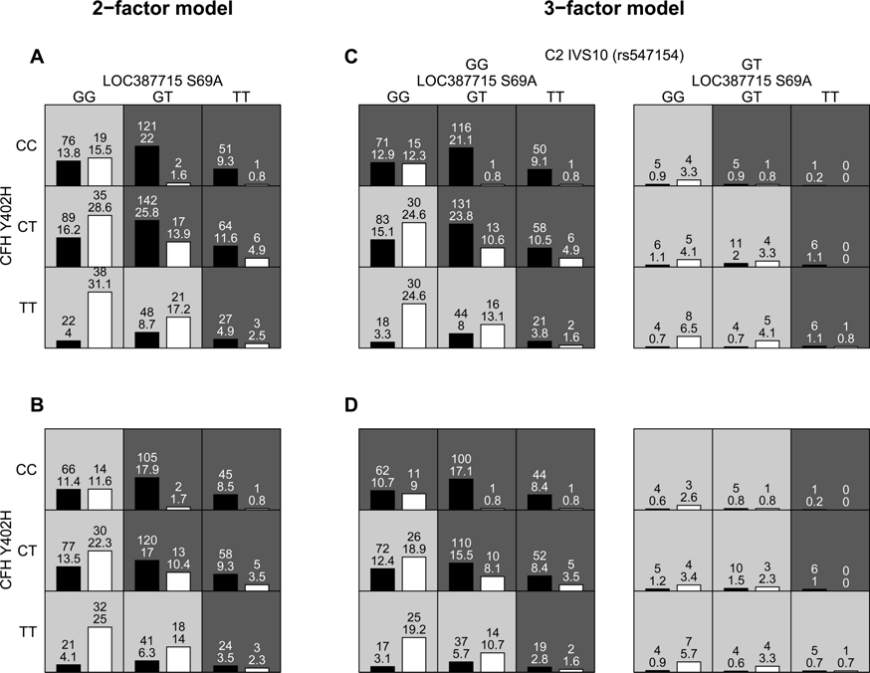
Results of GMDR analyses. A: Results of unadjusted GMDR analysis for the best two-factor model. B: Results of adjusted GMDR analysis for the best two-factor model. C: Results of unadjusted GMDR analysis for the three-factor model. D: Results of adjusted GMDR analysis for the three-factor model. Dark grey and light grey boxes correspond to the high- and low-risk genotype combinations, respectively. The black and white bars within each box correspond to cases and controls, respectively. The top number above each bar is number of individuals and the bottom number is the sum of scores for the corresponding group of individuals (cases or controls with particular three-locus genotype). The heights of the bars are proportional to the sum of scores in each group.

The two-factor GMDR unadjusted and adjusted models (Figure 2A and B) classify everyone with a homozygous (*TT)* LOC387715 risk genotype as cases and everyone with the homozygous (*GG*) *LOC387715* non-risk genotype as controls. On the other hand, individuals heterozygous (*GT*) at *LOC387715* need to have at least one *CFH* risk allele (*C*) to be classified as cases. When comparing the unadjusted two-factor model (Figure 2A) to the unadjusted three-factor model (Figure 2C), the most dramatic change is in the upper left most cell (*CC-GG CFH-LOC387715* joint genotype): 76 cases in that cell that were wrongly classified as controls by the two-factor model while 71 are correctly (5 wrongly) classified in the three-factor model. This increases the sensitivity from 63% to 73%, but comes at a cost of decreased specificity (80% to 72%).

Now looking more closely at the three-factor models (Figures 2C and D), the results of the GMDR analyses suggest that having at least one copy of the protective allele (*T*) at *C2*/*CFB* may reduce the risk contributed by *CFH* and *LOC387715* risk genotypes. For example in the unadjusted model (Figure 2C), individuals with the *CT-GT* and *CT-GG* two-locus genotypes at *CFH* and *LOC387715* and without the *C2*/*CFB* protective allele are classified as cases while those with the protective allele are classified as controls. In the adjusted model (Figure 2D), however, individuals with the *CT-GT* and *CC-GG* as well as *TT-TT* and *CC-GT* two-locus genotypes at *CFH* and *LOC387715*, are classified as controls if they carry the *C2*/*CFB* protective allele but cases otherwise. Note that the difference between the three-factor unadjusted and adjusted models is not due to the smaller dataset used in the adjusted analysis. To make sure this was not the case, we ran unadjusted analysis on the smaller dataset and arrived at the same model as in the unadjusted analysis. The predictive models presented in Figure 2 seem sensible as the predicted high-risk two- and three-locus genotypes group together. The predictive accuracy of the three-factor model measured by sensitivity, specificity, and balanced accuracy is >70% of both the unadjusted and adjusted models (Table 5). In the unadjusted analysis all five cross-validations suggest that the classification scheme classifies individuals significantly better than random (P-values <0.05) and in the adjusted analysis the classification is significantly better in all but one cross-validation experiment (Table 5). Both models provide excellent fit to the whole data (P-values <0.0001). In Table S2, we present the joint genotype and relative genotype frequencies in cases and controls, which provides a complementary view of the same findings as in Figure 2.

**Table 5:** Results of GMDR analyses.

		Unadjusted				Adjusted			
Model		P-value	Sensitivity	Specificity	Balanced Accuracy	P-value	Sensitivity	Specificity	Balanced Accuracy
CFH, LOC387715, and C2									
	Testing 1	0.0079	0.76	0.62	0.69	0.0029	0.70	0.79	0.74
	Testing 2	0.0140	0.55	0.79	0.67	0.0047	0.70	0.77	0.73
	Testing 3	0.0027	0.73	0.71	0.72	0.0172	0.75	0.64	0.70
	Testing 4	0.0001	0.65	0.93	0.79	0.0237	0.49	0.86	0.68
	Testing 5	0.0298	0.71	0.61	0.66	0.0823	0.75	0.54	0.64
	Average	…	0.68	0.73	0.71	…	0.68	0.72	0.70
	Whole data	<0.0001	0.73	0.72	**0.72**	<0.0001	0.70	0.74	**0.72**
CFH and LOC387715									
	Testing 1	0.0079	0.63	0.76	0.69	0.0026	0.66	0.83	0.74
	Testing 2	0.0140	0.55	0.79	0.67	0.0038	0.76	0.72	0.74
	Testing 3	0.0087	0.77	0.61	0.69	0.0320	0.62	0.73	0.68
	Testing 4	0.0003	0.67	0.86	0.76	0.0165	0.52	0.86	0.69
	Testing 5	0.0117	0.66	0.71	0.69	0.2298	0.75	0.45	0.60
	Average	…	0.66	0.75	0.70	…	0.66	0.72	0.69
	Whole data	<0.0001	0.63	0.80	**0.71**	<0.0001	0.61	0.80	**0.71**
CFH									
	Testing 1	0.0317	0.89	0.38	0.64	0.0276	0.88	0.45	0.66
	Testing 2	0.0341	0.78	0.52	0.65	0.0764	0.41	0.85	0.63
	Testing 3	0.1653	0.85	0.32	0.59	0.1413	0.83	0.39	0.61
	Testing 4	0.0011	0.83	0.64	0.74	0.1484	0.81	0.41	0.61
	Testing 5	0.0794	0.89	0.32	0.61	0.0400	0.89	0.41	0.65
	Average	…	0.85	0.44	0.64	…	0.77	0.50	0.63
	Whole data	<0.0001	0.85	0.44	**0.64**	<0.0001	0.85	0.45	**0.65**
LOC387715									
	Testing 1	0.0132	0.67	0.69	0.68	0.0564	0.71	0.60	0.66
	Testing 2	0.0447	0.67	0.62	0.65	0.0074	0.71	0.73	0.72
	Testing 3	0.0012	0.73	0.75	0.74	0.1565	0.72	0.51	0.61
	Testing 4	0.0305	0.74	0.57	0.66	0.0140	0.60	0.81	0.70
	Testing 5	0.0224	0.73	0.61	0.67	0.1102	0.68	0.59	0.63
	Average	…	0.71	0.65	0.68	…	0.68	0.65	0.66
	Whole data	<0.0001	0.71	0.65	**0.68**	<0.0001	0.68	0.64	**0.66**

Each testing set corresponds to 1/5 of the data. The same individuals are in each testing set across models and within type of analysis (unadjusted or adjusted). The individuals are not necessarily the same in the testing sets across type of analysis because of the smaller number of individuals that were available in the adjusted analyses compared to the unadjusted analysis (see the text for details). The average is the average over the five testing sets and the P-value corresponds to χ^2^ tests of fitting the models to the testing sets or the whole data.

#### Logistic regression vs. GMDR

In both the logistic regression and GMDR analyses, the best fitting one-factor model is the model with *LOC387715* only (Tables 4–5; in the logistic regression, the *LOC387715* model has the lowest AIC of all one-factor models and, in the GMDR results the *LOC387715* model has the highest balanced accuracy (in both the unadjusted and adjusted analyses). However, the difference between the *CFH* and *LOC387715* one-factor models is, very small, and, as the GMDR analyses show, the difference lies in the sensitivity and specificity rather than the overall balanced accuracy measure (Table 5). The three-factor model of *CFH*, *LOC387715*, and *C2*/*CFB* effects is implicated as the best model in both the regression and GMDR analyses (Tables 4–5). The logistic regression analyses suggest that accounting for *C2*/*CFB* effects significantly improves the two-factor model of *CFH* and *LOC387715* only (P-value 0.002). The GMDR analyses show that this improvement is due the increases sensitivity but the balanced accuracy increases only from 71% to 72% (Table 5). The GMDR analyses also suggest that adjusting for age, gender, and cigarette smoking does not dramatically improve the fit of the models. In fact, all models (1-, 2-, and 3-factor) have approximately the same balanced accuracy irrespective of whether adjustment is made (Table 5).

### Results of gene-cigarette smoking interaction analysis

Cigarette smoking does not significantly interact with any of the three variants investigated in our data. The p-values of LRTs are 0.24, 0.99, and 0.43 for *Y402H* in *CFH*, *S69A* in *LOC387715*, and *IVS10* in *C2*, respectively. For all three genes the most parsimonious models, according to AIC, are the models with only the additive gene effect and no smoking effect (results not shown).

## Discussion

We have replicated the association of one *C2* variant (*rs547154*) with ARM in both our case-control and family datasets and we have shown that accounting for the effects of *C2*/*CFB* significantly improves the fit of the logistic regression model in comparison to the two-factor model of joint additive effects of *CFH* and *LOC387715* (Table 2 and 4). Interestingly, both of the non-synonymous coding changes, *E318D* (*rs9332739*) in *C2* and *L9H* (*rs415667*) in *CFB*, identified by Gold et al. [60] are insignificant in both of our datasets. However, as these variants (*rs9332739* and *rs415667*) are quite rare, power to detect these variants is low. Even so, our independent confirmation of the statistically significant effect of this locus in ARM in two datasets, including family-based data, further supports the contribution of this locus to the genetic susceptibility of ARM.

As mentioned above, accounting for the effect of the *C2/CFB* locus significantly improved the fit of a logistic regression model of additive effects of *CFH* and *LOC387715* variants. To further understand this, we built predictive models of these three loci using the new generalized multifactor dimensionality reduction method (GMDR), and found that addition of C2/CFB to the model increased sensitivity (from 63% to 73%). However, the specificity is lowered (from 80% to 72%) and so the balanced accuracy only increases from 71% for the two-factor *CFH-LOC387715* model to 72% for the three-factor *CFH-LOC387715-C2* model in the unadjusted analysis (Table 5). If it were considered more important to identify cases than controls correctly, while maintaining a reasonable specificity, the three-factor model would be the better choice.

Since our associated variant (*rs547154*) in *C2/CFB* is rare, it is expected that accounting for the effect of this locus, using *rs547154* as a tag, would not markedly improve the overall prediction accuracy of the genetic risk model with *CFH* and *LOC387715* effects only, even though the effect may be strong. Although, positive associations in the *C2/CFB* region have been found and replicated primarily for rare variants [27], [60], [61], we cannot exclude the possibility that the true causal variant(s) in this region may be common, especially since not all known common SNPs have been typed in *C2/CFB* studies (Figure S1). Obviously, a genetic risk model of *CFH, LOC387715,* and *C2/CFB* effects could be quite different from our model presented here if the *C2/CFB* causal variant(s) were common, as then the rare *rs547154* would be a bad proxy.

Another concern regarding correctness of the three-factor model is the small sample size for the ‘protective’ *GT* genotype at *IVS10 (rs547154)* in *C2* (Figure 2 and Table S1), although it is important to remember that cross-validation does guard against over-fitting due to small sample sizes or a large number of parameters. The least stable classifications in Figure 2C are those cells in which the height of the bars is similar or number of individuals is low. In such cases, the classification rule can change if only a few individuals were added to that cell. For example, if we had only one additional control with a *CC-GG-GG CFH-LOC387715-C2* genotype (upper left most cell, left panel in Figure 2C), then individuals with this genotype combination would have been classified as controls instead of cases. To construct our original unrelated data set, we picked one case at random from each of the families. Figure S2 examines the sensitivity of our three-factor analyses when we randomly re-pick one case from each family. We created 10 other combined data-sets (overlap among cases from the families ranges from 57% to 66%) and ran the GMDR method. The figure clearly shows that only classifications corresponding to the rare *GT* genotype at *IVS10 (rs547154)* in *C2* are changed across samples, while the classifications corresponding to the common *GG* genotype are robust.

Accounting for covariates (age, gender, and cigarette smoking) failed to improve the prediction accuracy of the genetic risk models (Table 5). In fact, for the one- and two-factor models, the adjusted analyses arrived at the same high-risk (and low-risk) genotype combinations as the unadjusted analyses. The difference in sensitivity, specificity, and balanced accuracy between the two analyses is solely due to different number of individuals used in each set of analyses (because of incomplete smoking information). In the three-factor model, genotypes were grouped differently depending on whether unadjusted or adjusted analyses were performed (Figure 2) and, as mentioned in the results section, this difference is not solely due to different number of individuals used in each set of analyses.

The one-factor models of *CFH* and *LOC387715* did worse than the higher-factor models (balanced accuracy 64% and 68%, respectively), although, when considering they only model genetic effects at one locus, both models perform amazingly well. The GMDR method selected the *LOC387715* model as the best of all the one-factor models. However, depending on what the goals of using a prediction model are, one could easily choose the *CFH* model as the best one-factor model. For example, the sensitivity of the *CFH* model is much higher than of the *LOC387715* model (85% vs. 71%), but this increased sensitivity comes at a cost of low specificity (44% vs. 65%).

In their original report on the *C2*/*CFB* locus in ARM, Gold et al. [60] did not include *LOC387715* variants and, using a genetic algorithm search approach, they arrived at a genetic risk model of two *CFH* variants and three *C2*/*CFB* variants. The sensitivity and specificity of their model were 74% and 56%, respectively (which results in balanced accuracy of 65%). Interestingly, our three-factor model, which includes *LOC387715* effects, provides a better prediction accuracy (balanced accuracy 72%), similar specificity (73%), and better specificity (72%) than their more complicated five-factor model. Furthermore, even our simpler two-factor model of *CFH* and *LOC387715* effects also provides better prediction accuracy (balanced accuracy 71%).

We believe that a word of caution must be provided with regard to the possible use of these predictive models in clinical situations. It must be understood that the models presented in this paper and by others are based on comparison of extreme phenotypes (those with advanced forms of ARM and age-matched controls with minimal or no clinical findings). This does not address the determination of ARM risk for individuals for whom mild to moderate retinal findings are present. Secondly, odds ratios based on case-control association studies are not comparable to prospective, population-based relative risk assessments that still need to be done for ARM. Finally, one must always consider the composition of the population that may be subjected to molecular genetic screening. If we are considering the general population for whom the risk of ARM-related vision loss is less than 1% over their lifetime, then the current genetic models have inadequate levels of specificity to avoid a high percentage of false positive results. However, for individuals from high-risk cohorts for whom the prevalences of the high-risk variants are known, molecular diagnostic testing may be sufficiently discriminating of relative risk, though it is unclear how such knowledge would affect individual behavior or preventive treatments at this time.

In summary, we have confirmed the likely influence of the *C2*/*CFB* locus on ARM and shown that accounting for the effects at this locus can likely further stratify individuals as being at high or low risk of developing ARM. The important role the classical and/or alternative complement pathways seem to have in the disease-pathology of ARM should now encourage investigators to not only look at more complement pathway genes, but also to establish the biological mechanism behind the influence of *LOC387715* (or *HTRA1*) on the development of the disorder. Then, once either *LOC387715* or *HTRA1* has been convincingly shown to be the true ARM susceptibility gene on 10q26, it is likely that we will see similar trends in discoveries of genes involved in the same pathway as either of those genes.

## Supporting Information

Text S1 Logistic regression analyses
(0.04 MB PDF)

Text S2 Association analyses-*CFH* and *LOC387715*
(0.04 MB PDF)

Table S1 Genotype counts for *C2/CFB* variants, Y402H in *CFH*, and S69A in *LOC387715*
(0.04 MB PDF)

Table S2 Joint and relative genotype frequencies
(0.05 MB PDF)

Figure S1 Minor allele frequency of HapMap variants in the *C2/CFB* region
(0.06 MB PDF)

Figure S2 Sensitivity of three-factor GMDR model
(0.05 MB PDF)
