# Supplementary material for: C2 and CFB Genes in Age-Related Maculopathy and Joint Action with CFH and LOC387715 Genes
Source: PLoS One. 2008 May 21;3(5):e2199. doi: 10.1371/journal.pone.0002199 (PMC2374901; doi:10.1371/journal.pone.0002199)
Supplement: Text S1 — Logistic regression analyses (0.04 MB PDF) [file pone.0002199.s001.pdf]

Logistic regression analyses

Coding in two-factor models

A series of logistic regression models were fitted to the data in order to find a parsimonious model for the joint effects of each pair of loci. Models allowing for additive effects (ADD1, ADD2, and ADD-BOTH), models incorporating dominance effects (DOM1, DOM2, and DOM-BOTH), and three interaction models (ADD-INT, ADD-DOM, and DOM-INT) were fitted. In the additive models the genotypes, RR, RN, and NN (or PP, PN, and NN), are coded 1, 0, and 1, respectively; where R denotes the assumed risk allele at *CFH* or *LOC387715*, P the assumed protective allele at *C2*, and N the assumed normal allele. The dominance models incorporate a variable to the additive models coded as 0.5 for RR (or PP) and NN and 0.5 for RN (or PN). We let x_1 and x_2 denote the genotype variables in the additive models, and z_1 and z_2 the additional variables incorporated into the dominance models. Then the ADD1, ADD2, and ADD-BOTH models include terms (x_1), (x_2), and (x_1 and x_2), respectively, and the DOM1, DOM2, DOM-BOTH models incorporate terms (z_1), (z_2), and (z_1 and z_2) to the ADD1, ADD2, and ADD-BOTH models, respectively. Three further interaction models are fitted: ADD-INT incorporates the product term (x_1x_2) to the ADD-BOTH model, ADD-DOM incorporates the product terms ($x1x2$, $x1z2$, and $z1x2$), and DOM-INT incorporates the product terms (x_1x_2, x_1z_2, z_1x_2, and z_1x_2) to the DOM-BOTH model.

Coding in three-factor models

Since, for each pair of loci, the two-factor analyses implicated additive models as the most parsimonious and to keep the number of parameters as small as possible we only fit three-factor additive models without interaction. The models are ADD1, ADD2, ADD3, ADD12, ADD13, ADD23, and ADD123 and include terms (x_1), (x_2), (x_3),

(x_1 and x_2), (x_1 and x_3), (x_2 and x_3), and (x_1, x_2, and x_3), respectively, where x_1, x_2, and x_3, are coded as in the additive two-factor models above.
