# Supplementary material for: C2 and CFB Genes in Age-Related Maculopathy and Joint Action with CFH and LOC387715 Genes
Source: PLoS One. 2008 May 21;3(5):e2199. doi: 10.1371/journal.pone.0002199 (PMC2374901; doi:10.1371/journal.pone.0002199)
Supplement: Text S2 — Association analyses-CFH and LOC387715 (0.04 MB PDF) [file pone.0002199.s002.pdf]

Association analyses - *CFH* and *LOC387715*

In our prior studies, we tested the associations of Y402H in CFH [1] and S69A in LOC387715 [2] in a smaller subset of our data, than we have typed now. In our larger dataset both variants are highly associated with ARM (allelic and genotypic P-values < 0.00001 in both the case-control and family data, Table 2) providing further confirmation for the likely involvement of these genes in ARM pathogenesis. The ORs for individuals heterozygous and homozygous for the risk allele at Y402H are 4.11 (95% CI 2.28 to 7.40) and 8.96 (95% CI 4.49 to 17.88), and the corresponding PARs are 56% (95% CI 34% to 71%) and 53% (95% CI 31% to 69%), respectively. The ORs for individuals heterozygous and homozygous the S69A risk allele are 3.63 (95% CI 2.19 to 6.03) and 8.24 (95% CI 3.81 to 17.81), and the corresponding PARs are 42% (95% CI 26% to 54%) and 32% (95% CI 17% to 44%), respectively.

References

[1] Conley YP, Thalamuthu A, Jakobsdottir J, Weeks DE, Mah T, et al. (2005) Candidate gene analysis suggests a role for fatty acid biosynthesis and regulation of the complement system in the etiology of age-related maculopathy. Hum Mol Genet 14:1991–2002.

[2] Jakobsdottir J, Conley YP, Weeks DE, Mah TS, Ferrell RE, et al. (2005) Susceptibility genes for age−related maculopathy on chromosome 10q26. Am J Hum Genet 77:389–407.
