# Supplementary material for: C2 and CFB Genes in Age-Related Maculopathy and Joint Action with CFH and LOC387715 Genes
Source: PLoS One. 2008 May 21;3(5):e2199. doi: 10.1371/journal.pone.0002199 (PMC2374901; doi:10.1371/journal.pone.0002199)
Supplement: Table S1 — Genotype counts for C2/CFB variants, Y402H in CFH, and S69A in LOC387715 (0.04 MB PDF) [file pone.0002199.s003.pdf]

Table S1: Genotype counts for *C2/CFB* variants, *Y402H* in *CFH*, and *S69A* in *LOC387715*

					Allele labeling		Genotype counts in					
							Cases			Controls		
SNP	Gene	Chr	Bp[a]	Location[a]	1	2	11	12	22	11	12	22
rs9332739	*C2*	6	32011783	*E318D*	*C*	*G*	0	10	172	1	9	156
rs547154	*C2*	6	32018917	*IVS10*	*G*	*T*	170	9	0	130	31	0
rs4151667	*CFB*	6	32022003	*L9H*	*A*	*T*	0	10	168	1	10	156
rs2072633	*CFB*	6	32027557	*IVS17*	*A*	*G*	21	74	81	20	88	55
rs1061170	*CFH*	1	194925860	*Y402H*	*T*	*C*	21	80	60	69	64	22
rs10490924	*LOC387715*	10	124204438	*S69A*	*G*	*T*	50	74	40	103	42	10

Chr = chromosome

Bp = base pairs

[a] Bp and location within the genes are from NCBI build 127 (human genome build 36.2)
