# Supplementary material for: C2 and CFB Genes in Age-Related Maculopathy and Joint Action with CFH and LOC387715 Genes
Source: PLoS One. 2008 May 21;3(5):e2199. doi: 10.1371/journal.pone.0002199 (PMC2374901; doi:10.1371/journal.pone.0002199)
Supplement: Table S2 — Joint and relative genotype frequencies (0.05 MB PDF) [file pone.0002199.s004.pdf]

**Table S2** Joint and relative genotype frequencies

| | | | 2-factor model | | | 3-factor model | | | | | |
|---|---|---|---|---|---|---|---|---|---|---|---|
| | | | | | | C2 | | | | | |
| | | | | | | GG | | | GT | | |
| | | | LOC | | | LOC | | | LOC | | |
| | | | GG | GT | TT | GG | GT | TT | GG | GT | TT |
| Controls | CFH | CC | 0.1338 | 0.0141 | 0.0070 | 0.1056 | 0.0070 | 0.0070 | 0.0282 | 0.0070 | 0.0000 |
| | | CT | 0.2465 | 0.1197 | 0.0423 | 0.2113 | 0.0915 | 0.0423 | 0.0352 | 0.0282 | 0.0000 |
| | | TT | 0.2676 | 0.1479 | 0.0211 | 0.2113 | 0.1127 | 0.0141 | 0.0563 | 0.0352 | 0.0070 |
| Cases | CFH | CC | 0.1188 | 0.1891 | 0.0797 | 0.1109 | 0.1812 | 0.0781 | 0.0078 | 0.0078 | 0.0016 |
| | | CT | 0.1391 | 0.2219 | 0.1000 | 0.1297 | 0.2047 | 0.0906 | 0.0094 | 0.0172 | 0.0094 |
| | | TT | 0.0344 | 0.0750 | 0.0422 | 0.0281 | 0.0688 | 0.0328 | 0.0062 | 0.0062 | 0.0094 |
| Control/Case Ratios | CFH | CC | 1.13 | 0.07 | 0.09 | 0.95 | 0.04 | 0.09 | 3.62 | 0.90 | 0.00 |
| | | CT | 1.77 | 0.54 | 0.42 | 1.63 | 0.45 | 0.47 | 3.74 | 1.64 | 0.00 |
| | | TT | 7.78 | 1.97 | 0.50 | 7.52 | 1.64 | 0.43 | 9.08 | 5.68 | 0.74 |

The genotype frequencies are for the subset of our data used in the GMDR unadjusted analyses. This inlcudes individuals typed at all three loci (CFH, LOC387715, and C2). The cases include the unrelated cases and those randomly picked from the families (see the main text for details). The Control/Case Ratios are the ratios of the joint allele frequencies in controls versus cases. The gray-highlighted cells corrspond to cells with ratio < 1. Note, that those are the same cells that were classified as cases in the GMDR analyses.
