# Supplementary material for: C2 and CFB Genes in Age-Related Maculopathy and Joint Action with CFH and LOC387715 Genes
Source: PLoS One. 2008 May 21;3(5):e2199. doi: 10.1371/journal.pone.0002199 (PMC2374901; doi:10.1371/journal.pone.0002199)
Supplement: Figure S1 — Minor allele frequency of HapMap variants in the C2/CFB region (0.06 MB PDF) [file pone.0002199.s005.pdf]

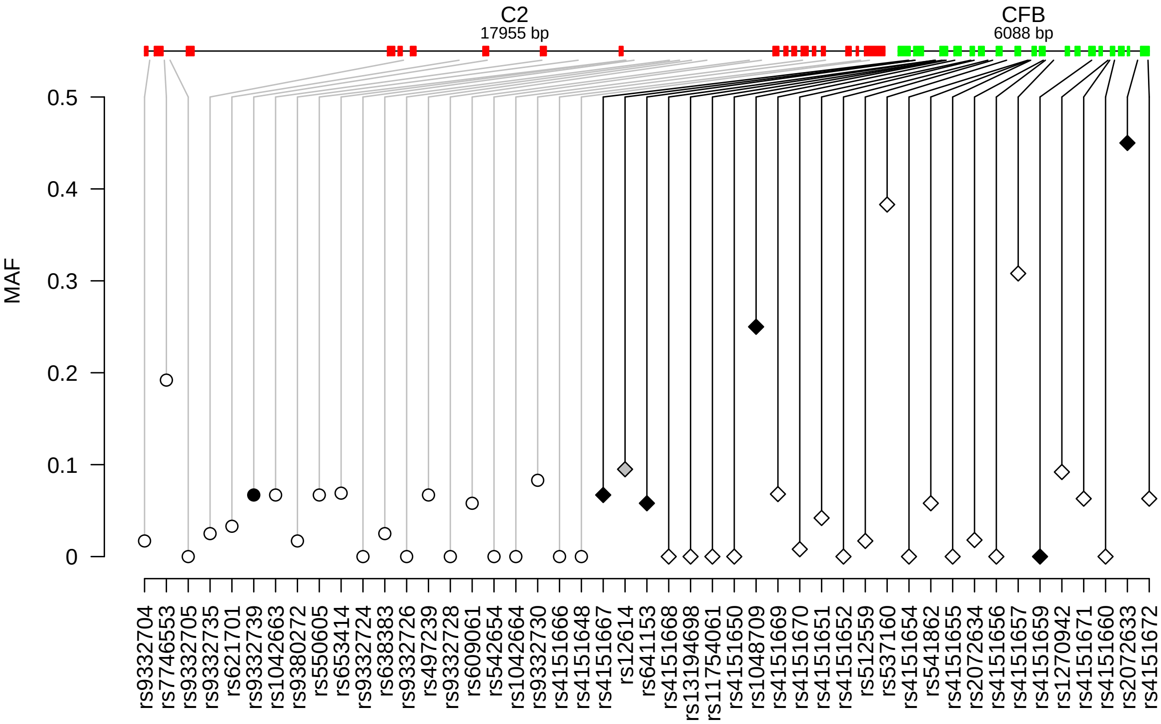

Figure S1: Minor allele frequency (MAF) of SNPs typed for the HapMap CEU population [1] in the *C2/CFB* region. Locations of the SNPs within the genes are shown. Red lines/boxes show the locations of exons in *C2* and green lines/boxes the locations of exons in *CFB*. White symbols represent SNPs not yet typed in any *C2/CFB* study [2, 3, 4] (including the present study), black filled symbols represent SNPs typed by Gold et al. [2] and grey filled symbols represent SNPs typed in *C2/CFB* study [4] other than the Gold et al. study [2]. Grey lines and circles correspond to SNPs in *C2* and black lines and dimonds correspond to SNPs in *CFB*.

References

[1] The International HapMap Consortium (2003) The International HapMap Project. Nature 426:789–796.

[2] Gold B, Merriam J, Zernant J, Hancox L, Taiber A, et al. (2006) Variation in factor B (BF) and complement component 2 (C2) genes is associated with age-related macular degeneration. Nat Genet 38:458–462.

[3] Maller J, George S, Purcell S, Fagerness J, Altshuler D, et al. (2006) Common variation in three genes, including a noncoding variant in CFH, strongly influences risk of age-related macular degeneration. Nat Genet 38:1055–1059.

[4] Spencer KL, Hauser MA, Olson LM, Schmidt S, Scott WK, et al. (2007) Protective effect of complement factor B and complement component 2 variants in age-related macular degeneration. Hum Mol Genet 16:1986–1992.
