# Supplementary material for: C2 and CFB Genes in Age-Related Maculopathy and Joint Action with CFH and LOC387715 Genes
Source: PLoS One. 2008 May 21;3(5):e2199. doi: 10.1371/journal.pone.0002199 (PMC2374901; doi:10.1371/journal.pone.0002199)
Supplement: Figure S2 — Sensitivity of three-factor GMDR model (0.05 MB PDF) [file pone.0002199.s006.pdf]

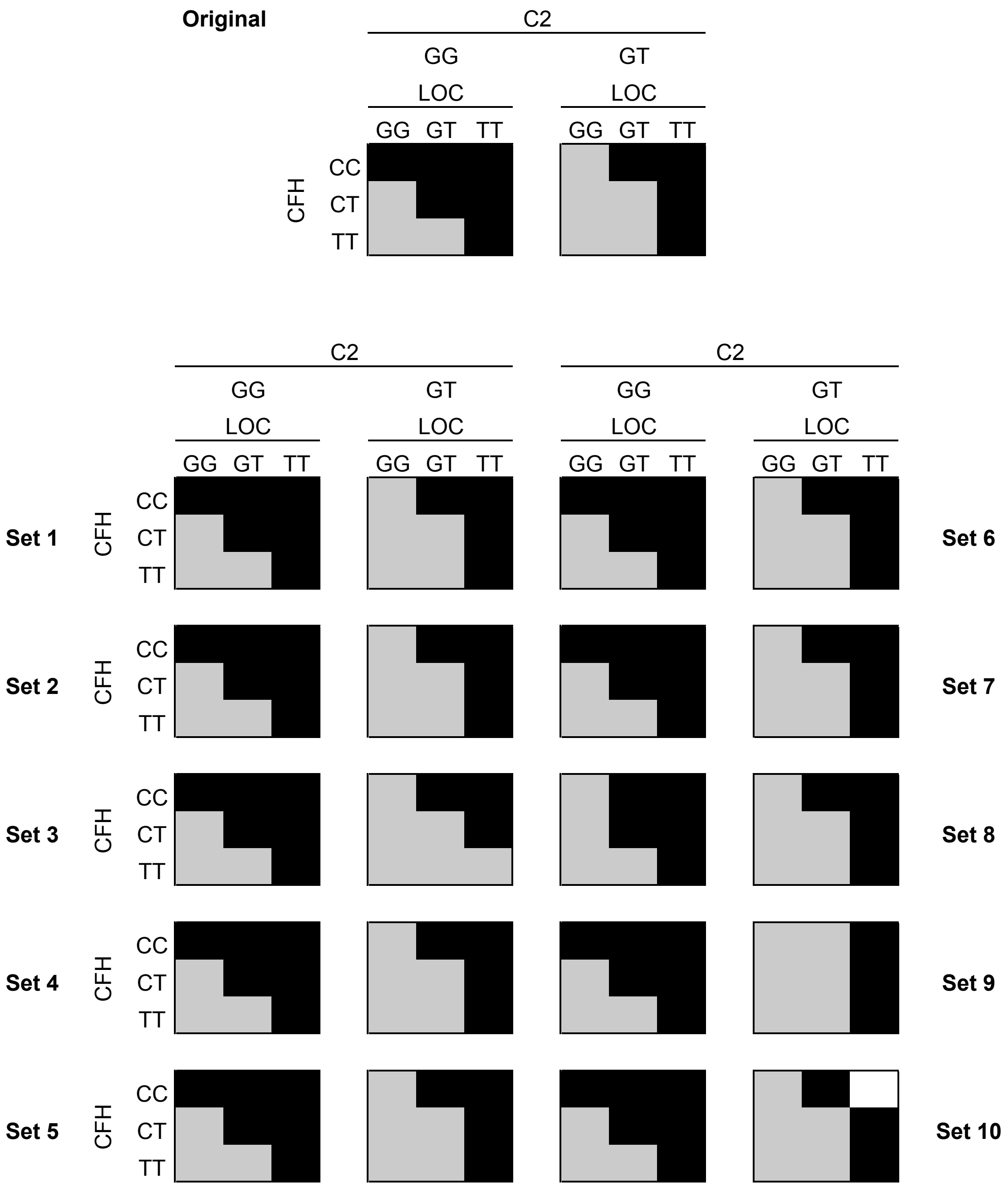

Figure S2: GMDR sensitivity analyses for the three-factor unadjusted model. The classification rules are shown for 10 data-sets, additional to the one used in the main paper (given at top). Each of those 10 data-sets has one case picked at random from each family. Black cells = cases, gray cells = controls, and white cells = empty cells/unknown status.
